# Influence of juvenile hormone analog on behavior in the red imported fire ant, *Solenopsis invicta*

**DOI:** 10.1038/s41598-023-41540-4

**Published:** 2023-09-07

**Authors:** Jesse Starkey, Chloe Hawkings, Cecilia Tamborindeguy

**Affiliations:** 1 Department of Entomology, Texas A&M University, 412 Heep Center, College Station, TX 77843 USA; 2 Present Address: Department of Entomology, Rutgers University, Thompson Hall, New Brunswick, NJ 08901 USA

**Keywords:** Ecology, Evolution

## Abstract

Division of labor is a hallmark characteristic of social insect colonies. While it is understood that worker differentiation is regulated through either the queen or her brood, the understanding of the physiology behind task regulation varies within social species. Studies in eusocial insects have shown that juvenile hormone (JH) is associated with division of labor and the onset of foraging tasks. Although, outside of a few key species, this interaction has yet to be elucidated in the red imported fire ant, *Solenopsis invicta*. In this study, we evaluated the role of a JH analog, S-hydroprene in worker task transition in *Solenopsis invicta*. S-hydroprene was applied to nurses to observe behavioral changes. S-hyroprene application to nurses did not affect phototaxis, but there was a shift in behavior from internal, nest-based behaviors to external, foraging-based behaviors. These results show that JH may be implicated in worker task transition in *S. invicta* and may function similarly as it does in other eusocial insects.

## Introduction

Division of labor occurs when individuals within a colony perform certain tasks associated with growth and maintenance of the whole colony. It is considered a hallmark characteristic of social insect structure^1^. Distinctions of task allocation can sometimes be indicated by polymorphic individuals that have morphological characters distinct to a specific caste. Social insects can also be monomorphic where the differences between individuals performing different tasks can be physiologically based and associated with nutrition, colony needs and age polyethism determine the task that is performed by workers^2,3^. Understanding the physiological basis of division of labor can provide insight on the evolutionary trajectory of eusocial colony structure.

In insects, juvenile hormone (JH) is typically associated with metamorphosis, development and reproduction^4^. However, there has been much research among both solitary and social insects, especially that of insects within the order Hymenoptera, that show its role in behavioral transition and task determination in insects^4–8^. In the honey bee *Apis mellifera*, high JH titers are associated with foraging behavior while low titers are associated with nursing behavior^8,9^. In some ant species such as *Pogonomyrmex californicus*, *Myrmicaria eumenodies*, and *Harpegnathos saltator*, JH titers were significantly higher in ants displaying extranidal behavior such as foraging and nest defense^10,11^, and topical applications of JH or JH-analogs induced foraging-like physiological changes^12^.

The red imported fire ant, *Solenopsis invicta*, provides a good model to further study the hormonal basis of work task transition, specifically the correlation of JH on task transition. The worker caste is comprised of sterile females which perform all tasks necessary for colony survival, growth, and maintenance^13^. For *S. invicta*, the most well-known aspect of JH is its function in queen development and behavior, often reflecting the reproductive status of the individual queens and their social form^14–16^. However, the role of JH in *S. invicta* workers remains understudied. Previously, the effect of the application of the JH analog S-hydroprene on the expression of genes such as *vitellogenin* and *hexamerin*, which are possibly co-opted for task allocation in workers, was evaluated. The application of JH analog to nurses caused shifts in expression of hexamerins to be similar to that of a forager, indicating a possible role in the shift from nurses to foragers^17,18^. However, these studies did not evaluate if the application also resulted in changes in the behavior of the ants.

In this study, we provide a behavioral assay to evaluate the prolonged effect of the application of the JH analog, S-hydroprene, on worker behavior, specifically in medium nurses. Nurses were treated with the analog or shams for a 7-day period, and the effect of the application was observed on phototaxis preferred placement in a microcolony (nest-like or foraging-like).

## Materials and methods

### Fire ant colony maintenance

Colonies were obtained from the Dale Watts Cross Country Course behind the Urban Entomology building on the Texas A&M campus (30° 37′ 12.7″ N 96° 22′ 09.6″ W) from January to March 2020. The colonies were kept in plastic trays coated with Fluon (Insect-a-slip, BioQuip products, CA, USA) and maintained at 27 ± 2 °C in a 12:12 h light/dark photoperiod. The colonies were fed daily with a 20% honey water solution and crickets, *Acheta domestica*, as well as being provided with water ad libitum.

Medium nurse ants were selected based on their head measurements as described in previous studies^19^. Medium workers were used in this study because they exhibit more behavioral flexibility compared to minor and majors, as well as more stability over time in the task they perform^20^. Ants in the nest directly interacting with the brood were considered nurses.

### JH-analog applications

Medium sized nurses were collected from the colonies and treated with 1 µl of S-hydroprene (diluted at 25 ng/µl dissolved in acetone), 1 µl of acetone, or sham-treated on the dorsal portion of the abdomen of the insect. For sham treatments, the method of JH analog application was mimicked on ants, however the pipette was empty. Following treatment, each ant was housed individually within a glass test tube stoppered with cotton and provided honey water and water during this time. Over a 7-day period, these ants were dosed again every other day at the same time for a total of 4 doses and returned to their test tube after each dose. In total, the experiment was performed 5 times: each replicate was performed using insects collected from a different colony (there were 9 or 10 ants per treatment in each replicate due to mortality). The rate of mortality after JH analog or acetone application was 6% for each treatment.

### Phototaxis experiment

**Figure 1:**
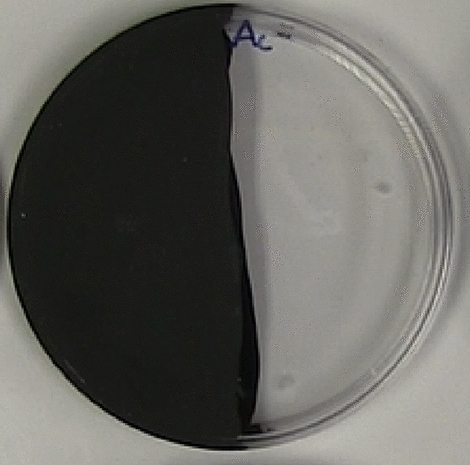
Petri dish used for the phototaxis bioassay after treatment.

To determine if the topical application of the JH-analog altered worker phototaxis, after the 7-day treatment the workers were placed individually into a 4-inch petri dish half covered with black paint applied on the outside top and bottom of the petri dish. The paint was applied to the outside to ensure that the workers never interacted with the paint (Fig. 1)^21^. Workers were placed gently in the center of the petri dish for each bioassay. Workers were given 5 min to habituate to the conditions within the petri dish before recording. After 5 min, the workers were recorded with a camera, observing, and tracking the time spent in the light portion of the petri dish for 10 min. After 10 min, the workers were removed and placed within a microcolony with the other workers from the same treatment group (there were 9 or 10 ants per treatment in each replicate due to mortality).

### Microcolony bioassay

**Figure 2:**
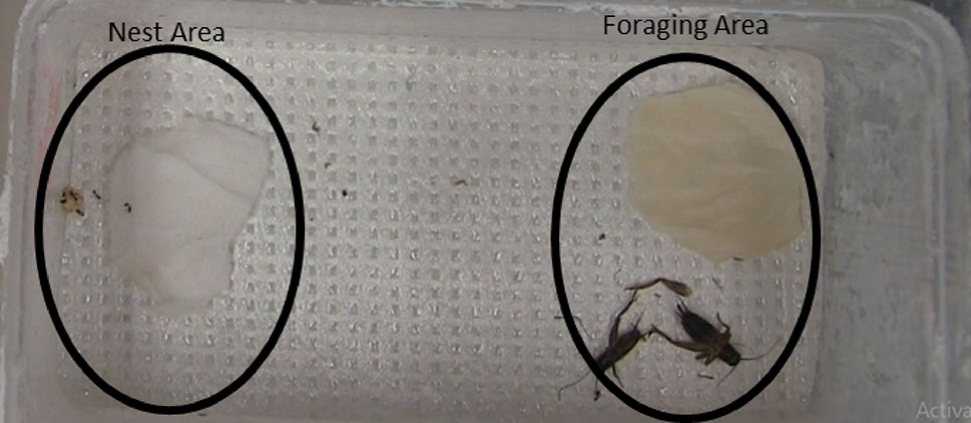
Microcolony setup for the behavioral assay associated with brood tending behavior in treated workers. Black circles indicate the areas within the microcolony that were considered nest-like and foraging-like conditions.

To discern if there were behavioral changes associated with the JH-analog treatments, workers were grouped into microcolonies based on their treatment groups (because of mortality there were 9 or 10 ants per microcolony for a total of 47 ants per treatment). The experiment was performed 5 times. Each microcolony contained crickets, 20% honey water, and 0.015 g of brood placed on a damp cotton pad. Brood was taken from the same colony as the workers and had an equal distribution of eggs, larvae, and pupae. The food and the brood were placed on opposite ends of the microcolony. Microcolonies were defined as a 12 × 6 cm plastic container coated with fluon to prevent escape, with an ample supply of protein and carbohydrate food sources on one end and a moist cotton to prevent desiccation and a spot to house brood on the other. The bottom of the microcolonies were covered with a non-stick lab bench protector paper. Once the workers were placed in the microcolony, they were allowed to acclimate to the new conditions for 24 h. After 24 h, cameras were set up to record the microcolonies for 8 h, observing the presence of workers within the microcolony every 30 min to determine whether the ants were on the brood side or the food side. Foraging- and nest-like conditions were determined by the position of the ants on top of cotton with carbohydrate and protein sources on the foraging side and the moist cotton with brood on the on the nest side of the microcolony to control random wandering and difficulty determining the location of workers on the border between each side (Fig. 2).

### Statistical analysis

Data was collected and displayed as means with standard error bars. Data normality distribution was determined by a Chi-squared goodness-of-fit test. The results of the phototaxis and microcolony bioassays were analyzed using a Kruskal–Wallis Ranked Sums test with a Wilcoxon Each Pair post hoc analysis when necessary. JMP Pro 16 was used for all statistical tests, considering *p*-values of less than 0.05 as significant.

## Results

### Phototaxis experiment

**Figure 3:**
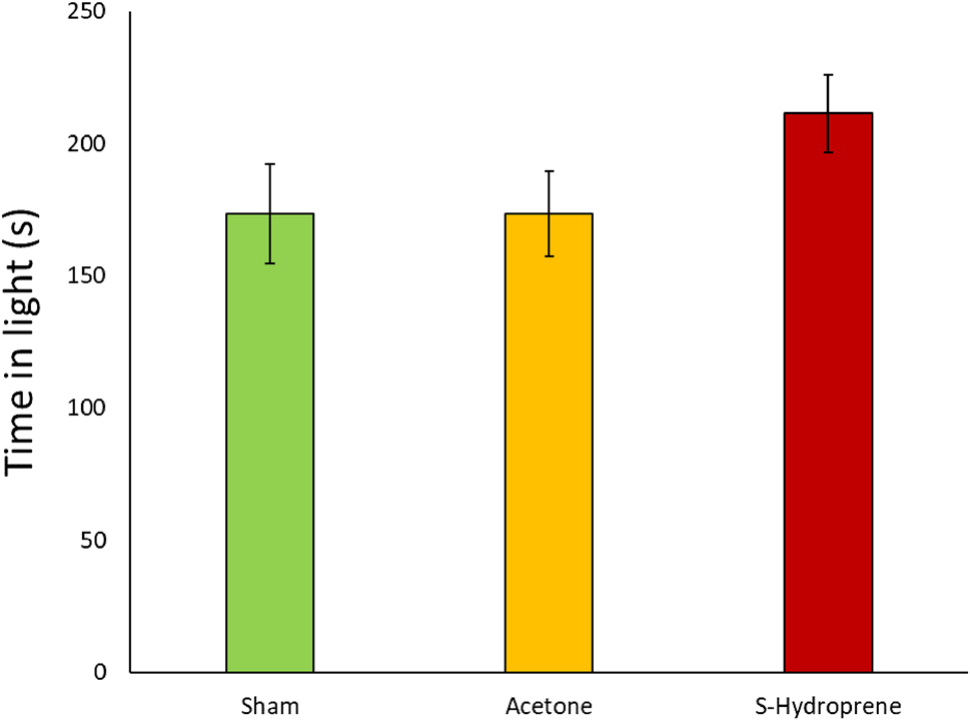
Time spent in light by treated workers (*n* = 47/treatment with 9 or 10 ants per treatment in each replicate due to mortality) during a 10-min observation period to determine differences in phototaxis after 5 treatments with S-hydroprene, acetone, or sham over a 7-day period (*p* = 0.1530). The bars represent the standard error of the mean.

The amount of time medium workers spent in the light when given a choice after 5 successive applications of the S-hydroprene, acetone, or sham treatment over a 7-day period was recorded. There was no significant difference between any of the three treatments (χ^2^ = 3.7547, df = 2, *p* = 0.1530) (Fig. 3).

### Microcolony bioassay

**Figure 4:**
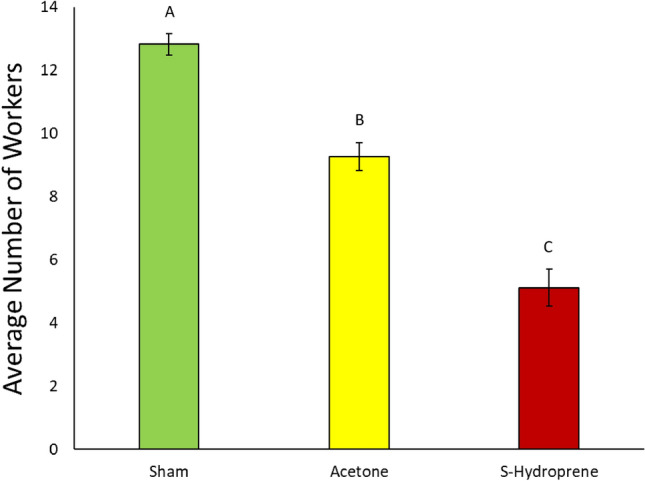
Average number of workers present in nest conditions observed over an 8-h period at 30-min snapshots (*n* = 47/treatment with 9 or 10 ants per treatment in each replicate due to mortality). Bars represent standard error of average number of workers found in nest conditions. Letters denote significance at *p* < 0.0001. The bars represent the standard error of the mean.

**Figure 5:**
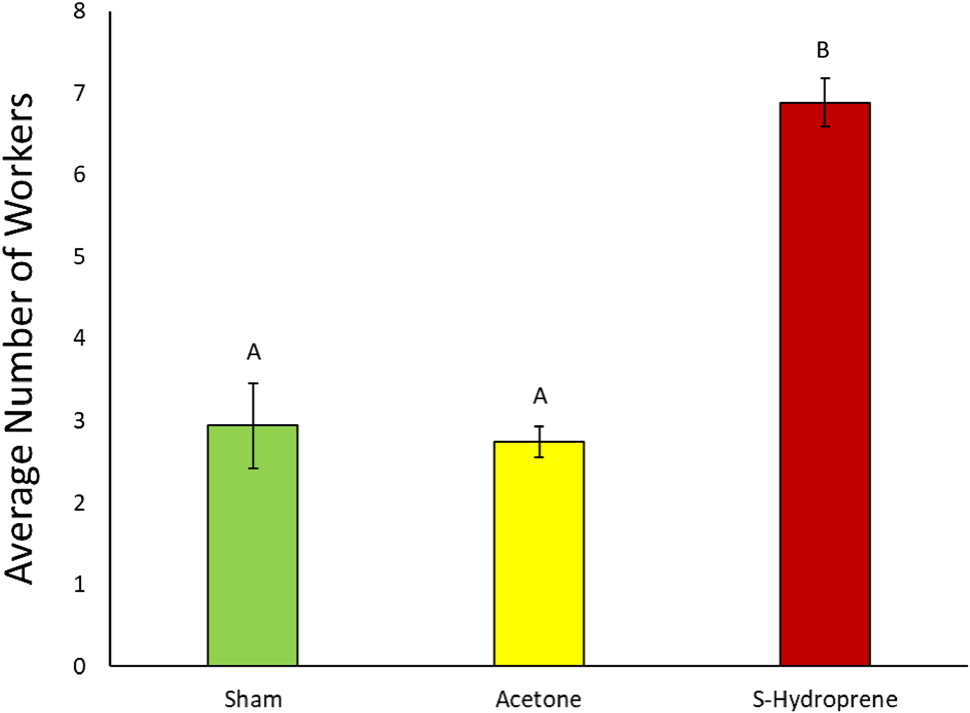
Average number of workers present in foraging conditions observed over an 8-h period at 30-min snapshots (*n* = 47/treatment with 9 or 10 ants per treatment in each replicate due to mortality). Bars represent standard error of average number of workers found in foraging conditions. Letters denote significance at *p* < 0.0001. The bars represent the standard error of the mean.

The average number of workers found in nest and foraging conditions over an 8-h observation period were calculated. Overall, there was a significant relationship between treatment and the position of the workers throughout the foraging bioassay (Nest Conditions: χ^2^ = 67.6263, df = 2, *p* < 0.0001; Foraging Conditions: χ^2^ = 46.0139, df = 2, *p* < 0.0001). There were significantly more workers found in nest conditions in groups treated with acetone or sham compared to S-hydroprene treated groups (S-Hydroprene versus Acetone: *Z* = − 6.12284, *p* < 0001; S-Hydroprene versus Sham: *Z* = − 6.77520, *p* < 0.001) (Fig. 4). There was a significantly higher number of workers found within nest conditions in the sham treatment compared to workers treated with acetone (Acetone versus Sham: *Z* = 4.62265, *p* < 0.0001) (Fig. 4). Additionally, there were significantly more workers found in foraging conditions in groups treated with S-hydroprene compared to both acetone groups and workers left untreated (S-Hydroprene versus Acetone: *Z* = 6.07514, *p* < 0001; S-Hydroprene versus Sham: *Z* = 5.095633, *p* < 0.001) (Fig. 5).

## Discussion

In insects, juvenile hormone titers are important in the development and reproduction of both solitary and social insects^4,22^. The results from our study show that the JH analog can cause a behavioral shift in *S. invicta* workers. JH has been shown to influence worker behavior and task in many other insect species, however the extent of this influence has yet to be fully understood.

Our results show that the direct application of the JH analog does not influence worker phototaxis or preference to light (Fig. 3). In some ant species, phototaxis can be used as a proxy for foraging. For example, in the study of the effect of JH in the behavior of *Acromyrmex octospinosus* workers, an increased attraction towards light was tied to behaviors relating to leaving the nest, typically that of foraging^23^. However, *S. invicta* workers are more influenced by temperature than light when it comes to foraging^24,25^. Therefore, the results of the phototaxis assay paired with that of the behavioral assay in the microcolonies indicate that light plays a small role if any in influencing foraging in *S. invicta*, as more workers were found within foraging conditions when treated with S-hydroprene compared to sham or acetone treatments (Fig. 5).

Several factors such as age or nutritional status of the worker could affect its propensity to switch from nursing to foraging. Indeed, it is known that age in eusocial insects like *Bombus impatiens*^26^, *A. mellifera*^3^ and even in *S. invicta*^27^ plays a role in the behavior the insect performs. In this study, the age of the workers was not controlled, instead, nurse ants interacting with the brood were randomly selected to perform the experiment. Despite this, our results show that the effect of the application of a JH analog on *S. invicta* worker behavior and task was consistent with those found in other ant, wasp, bee, and termite species when the insects were treated with a JH analog^7,8,10,11,28^. Future experiments could assess whether these other factors influence the effect of the juvenile hormone analog on the behavior of fire ant workers.

Overall, this study supports the notion that the role of JH might be conserved in *S. invicta* as a major regulator for division of labor.

## Data Availability

All data generated or analyzed during this study are included in this published article.
